# Modeling of networks and globules of charged domain walls observed in pump and pulse induced states

**DOI:** 10.1038/s41598-018-22308-7

**Published:** 2018-03-06

**Authors:** Petr Karpov, Serguei Brazovskii

**Affiliations:** 1 National University of Science and Technology “MISiS”, Moscow, Russia; 2 CNRS UMR 8626 LPTMS, University of Paris-Sud, University of Paris-Saclay, Orsay, France; 3 Jozef Stefan Institute, Jamova 39, Ljubljana, SI-1000 Slovenia

## Abstract

Experiments on optical and STM injection of carriers in layered MX_2_ materials revealed the formation of nanoscale patterns with networks and globules of domain walls. This is thought to be responsible for the metallization transition of the Mott insulator and for stabilization of a “hidden” state. In response, here we present studies of the classical charged lattice gas model emulating the superlattice of polarons ubiquitous to the material of choice 1*T* − TaS_2_. The injection pulse was simulated by introducing a small random concentration of voids which subsequent evolution was followed by means of Monte Carlo cooling. Below the detected phase transition, the voids gradually coalesce into domain walls forming locally connected globules and then the global network leading to a mosaic fragmentation into domains with different degenerate ground states. The obtained patterns closely resemble the experimental STM visualizations. The surprising aggregation of charged voids is understood by fractionalization of their charges across the walls’ lines.

## Introduction

Major anticipations for the post-silicon electronics are related to materials which demonstrate a layered structure with a possibility for exfoliation down to a few and even a single atomic layer, akin to the graphene. The latest attention was paid to oxides and particularly di-halcogenides of transition metals *MX*_2_ with *M* = Nb, Ta, Ti and *X* = S, Se, see e.g^1,2^. for reviews.

These materials show a very rich phase diagram spanning from unconventional insulators of the so called Peierls and Mott types to the superconductivity^3^. The transformations among these phases involve formation of superstructures like several types of so called charge density waves (CDW) and/or of hierarchical polaronic crystals. Recent studies of these materials fruitfully overlapped with another new wave in solid state physics. This is the science of controlled transformations of electronic states or even of whole phases by impacts of strong electric fields and/or the fast optical pumping. A super goal is to attend “hidden” states which are inaccessible and even unknown under equilibrium conditions. In relation to this article subjects, the success came recently from observations of ultrafast (at the scale of picoseconds) switching by means of optical^4,5^ and voltage^6,7^ pulses, as well by local manipulations^8,9^. The registered ultrafast switching is already discussed as a way for new types of RAM design, see^10^ and rfs. therein.

Most challenging and inspiring observations have been done in studies by the scanning tunneling microscopy (STM) and spectroscopy (STS)^5,8,9,11^. They have shown that the switching from an insulating to a conducting state proceeds via creation of local globules or extended networks of domain walls enforcing fragmentation of the insulating electronic crystal into a conducting mosaics of domains with different multiply degenerate ground states.

Most intriguing observations have been done upon very popular nowadays layered material 1*T* − TaS_2_ which is a still enigmatic “polaronic Wigner-crystalline Mott insulator”. The rich phase diagram of 1*T* − TaS_2_ includes such states as incommensurate, nearly commensurate, and commensurate CDWs which unusually support also the Mott insulator state for a subset of electrons. Recently, new long-lived metastable phases have been discovered: a “hidden” state created by laser^4,5^ or voltage^6,7^ pulses, and a most probably related “metallic mosaic” state created locally by STM pulses^8,9^.

1*T* − TaS_2_ is a narrow-gap Mott insulator existing unusually on the background formed by a sequence of CDW transitions which have gaped most of the Fermi surface of the high temperature metallic (with 1 electron per Ta site) parent phase^2^. Incomplete nesting leaves each 13-th electron ungaped which in a typical CDW would give rise to a pocket of carriers. Here, each excess carrier is self-trapped by inwards displacements of the surrounding atomic hexagon (forming the “David star” unit) which gives rise to the intragap local level accommodating this electron. Exciting the self-trapped electron from the intragap level leads to a partial relaxation of deformation, the David star levels out in favor of a void in the crystal of polarons. The charged voids are expected to arrange themselves into a Wigner crystal subjected to constraints of commensurability and packing with respect to the underlying structure.

A major question arises: why and how the repulsive voids aggregate into the net of walls leaving micro-crystalline domains in-between? In this paper, we answer this and related questions by modeling the superlattice of polarons upon the 2D triangular basic lattice of all Ta atoms by a classical charged lattice gas with a screened repulsive Coulomb interaction among the particles. The external pulse injecting the voids was simulated by introducing a small random concentration of voids reducing the particles concentration *ν* below the equilibrium *ν*_0_ = 1/13 (some other experimentally relevant concentrations are briefly described in the Supplementary Material, Sec. IV). The subsequent evolution of the system, including the passage through the thermodynamic first order phase transition, was studied by means of the Monte Carlo simulation. Surprisingly, this minimalistic model is already able to capture the formation of domain walls in a close visual resemblance with experimental observations and also to explain the effect qualitatively as an intriguing result of the charge fragmentation.

It is worth noticing that the described here phenomena can be viewed in a more general perspective of “nanonetworks” which notion unifies observations on nanosize aggregates in biological, physics and chemistry and the mathematical graphs theory, see the reviews^12,13^.

## The model

We model the system of polarons by a lattice gas of charged particles on a triangular lattice. Each particle represents the self-trapped electron in the middle of the David star, thus the effective charge is −*e*, which in average is compensated by the static uniform positive background.

**Figure 1:**
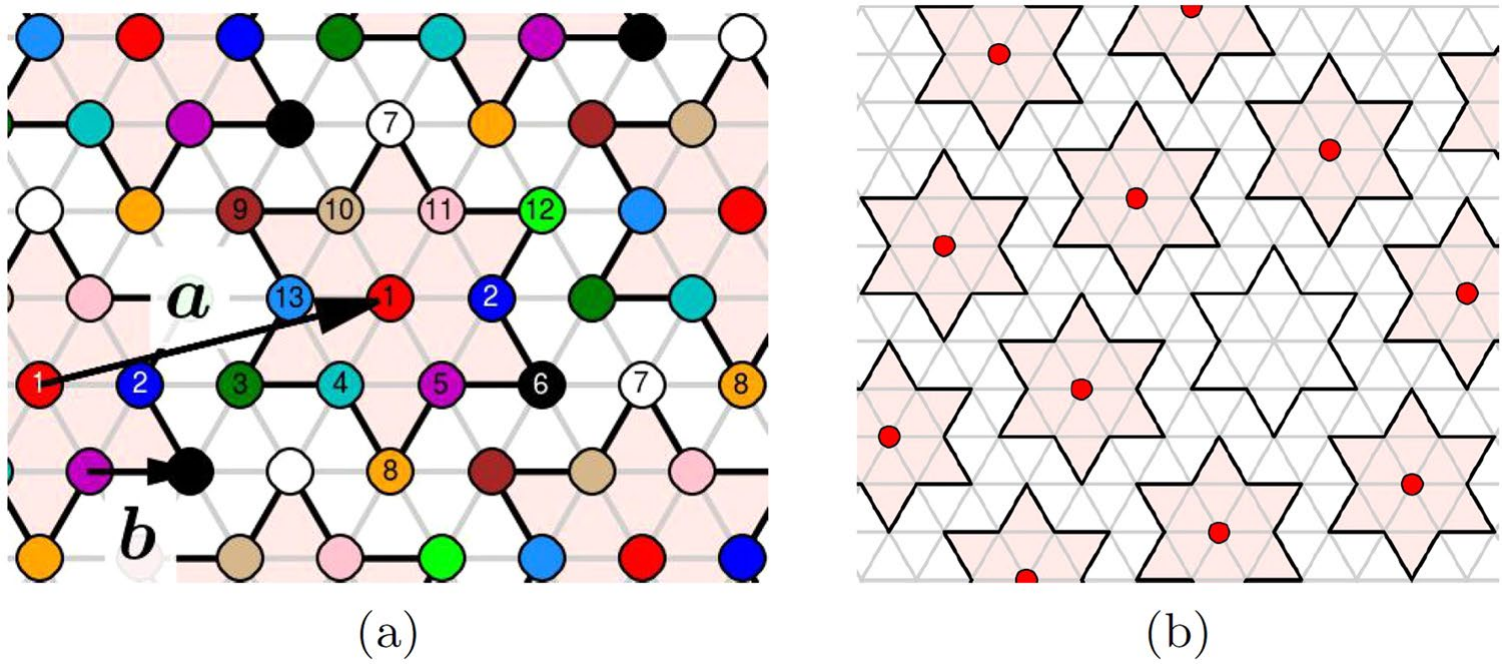
(**a**) Underlying triangular lattice (with the period *b*) of all Ta-atoms and the coloring scheme showing its 13 sublattices; in the ground state only one sublattice (with the period *a*) is occupied. The “right” chirality is shown; for the “left” chirality vector a would be reflected with respect to the line 1-2-3-4. (**b**) Lattice with *ν* = 1/13 concentration of polarons at presence of one void (the emptified “David star”). The occupied polaronic sites, marked by red circles, are surrounded by filled “David stars” which perimeters pass through neighbors which positions are actually displaced inwards (not shown here).

The external pulse is simulated by a small concentration of randomly seeded voids reducing the particles’ concentration below the equilibrium: *ν* = *ν*_0_ − *δν*. The interaction of polarons located at sites *i*, *j* is described by an effective Hamiltonian *H* = ∑_*i*,*j*_*U*_*ij*_*n*_*i*_*n*_*j*_ with repulsive interactions *U*_*ij*_. Here the sum is over all pairs of sites *i* ≠ *j*; *n*_*i*_ = 1 (or 0) when particle is present (or absent) at the site *i*, and we choose *U*_*ij*_ as the screened Coulomb potential

1 $${U}_{ij}=\frac{{U}_{0}a}{|{{\bf{r}}}_{i}-{{\bf{r}}}_{j}|}\exp (-\frac{r-a}{{l}_{s}}),$$

where *U*_0_ = *e*^2^ exp(−*a*/*l*_*s*_)/*a* is the Coulomb energy of interaction of particles at neighboring sites in the Wigner crystal state with the distance $a=\sqrt{13}b$ between them (*b* is the lattice spacing of the underlying triangular lattice, Fig. 1a), *l*_*s*_ is the screening length. The screening length comes from a small density of states at the Fermi level^8^. Considering the delocalized electrons as a 2D Fermi gas, we get ${l}_{s}\sim \sqrt{\pi \mathrm{/2}{d}_{3}{r}_{B}}$ where *d*_3_ is the interplane distance and *r*_*B*_ is the effective Bohr radius.

## Superlattice and its charged defects

In the ground state, the charged particles living on the triangular lattice tempt to arrange themselves in also the triangular superlattice, which is close-packed and most energetically favorable in 2D^14^ (with some notable exceptions for more exotic potentials^15^). Figure 1a illustrates, in accordance with all structural experiments, that it can be done indeed, with the superlattice vectors inclined by ±13.9° with respect to the host one–the chirality sign determining the mirror symmetry breaking (the right chirality was chosen to be shown.) The most important for us is the translational symmetry breaking leaving 13-fold degeneracy with respect to positioning of the David stars’ central atoms at the host sites. The total degeneracy is 26-fold. Until recently it was thought that the two mirror-symmetric phases do not not coexist within a given sample experimentally^16^ and in the modeling for the sufficiently slow cooling rates (probably because of the high energy of the corresponding twinning wall). However, recent experiments^17^ and the numerical modeling for the fast cooling rates indicate that the two chiral phases can coexist–see Supplementary Sec. V for the details.

**Figure 2:**
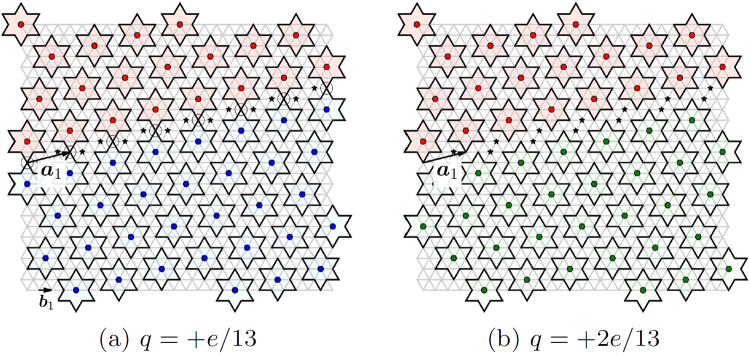
Positively charged domain walls with charges per unit cell length a: (**a**) +*e*/13; (**b**) +2*e*/13. The whole sequence of domain walls can be obtained by consecutive displacements of the blue domain by the vector b_1_ as indicated in (**a**). In (**a**), the sites are encircled where David stars within the wall share the corners. Black asterisks designate the sites not belonging to any star.

The David star, taken as a symmetric unit cell, contains three nonequivalent cites: the central one, the 6 nearest neighbors and 6 next-nearest neighbors. We shall associate the polaron location with the central site while basic allowed defects correspond either to its elimination (the void as the point defect) or to its replacement to one of NN or NNN positions (the domain walls as line defects). The simplest lattice defect is a void or a “polaronic hole” (Fig. 1b) which is formed when the electron from the intragap level is taken away or excited to the conduction band and soon the associated lattice distortions are partially released. The single void has the relative charge +*e* (keeping in mind the background neutralizing charge) and the Coulomb self-energy of the order ${E}_{void}\simeq {e}^{2}/a$. While the void is a particular manifestation of a general notion of vacancies in crystals, in our case there can be also a specific topologically nontrivial defect–the domain wall separating domains with a different 13-fold positional degeneracy of the ground state (Fig. 2). The domain wall cross-section resembles the discommensuration known in CDW systems^18^.

Experimentally, the lattice defects can be introduced via external pulses, by impurity doping or by the field effect. For example, a laser or STM pulse can excite the Mott-band electrons residing in the centers of the David star clusters, creating an ensemble of voids. Since the voids are charged objects, then at first sight they should repel each other and form a Wigner crystal themselves. But our modeling consistent with the experiment shows that the voids rather attract one another at short distances and their ensemble is unstable towards formation of domain walls’ net. Qualitatively, this instability can be understood from the following argument. Compare energies of the isolated void and of the domain wall segment carrying the same charge. The minimal charge of domain wall per the translation vector a_1_ is +*e*/13 (Fig. 2a), and the energy of the wall’s segment carrying the charge +*e* can be estimated as for a uniformly charged line:

2 $${E}_{wall}\simeq 13\times \frac{{(e\mathrm{/13)}}^{2}}{a}\,\mathrm{ln}({l}_{s}/a),$$

For moderate screening lengthes *l*_*s*_, it is lower than the void’s self-energy *E*_*void*_, making energetically favorable to decompose the voids into fractionally-charged domain walls. The local effects beyond our model can also favor domain walls with other charges: for the single-step +1*e*/13 domain wall there are anomalous sites where David stars intersect (Fig. 2a) which raises its energy and can make the double-step +2*e*/13 domain walls (Fig. 2b) to be energetically favorable. The same qualitative arguments can be applied to the case of doping by interstitials rather then voids.

## Numerical modeling

We simulated the cooling evolution of the classical lattice gas with the interaction potential (1) via Metropolis Monte Carlo algorithm (see Methods). We perform slow cooling from *T* = 0.055*U*_0_, which is above the detected ordering phase transition (see below), down to *T* = 0.01*U*_0_ with a step Δ*T* = −0.0001*U*_0_, reaching either a ground state or a very close in energy metastable state. Below we, first, consider undoped systems (where particles concentration *ν* is exactly *ν*_0_ = 1/13), and then systems doped by voids (with *ν* = *ν*_0_ − *δν* ≡ *ν*_0_(1 − *ν*_*voids*_), where *ν*_*voids*_ = *δν*/*ν*_0_ is the voids’ concentration). Results for another sign of doping are briefly presented in the Supplementary Material, Sec. IV.

### Undoped system

As a reference system we chose the sample with 91 × 104 sites with the total a number of particles *N*_*p*_ = 728, which corresponds to the concentration *ν*_0_ = 1/13.

**Figure 3:**
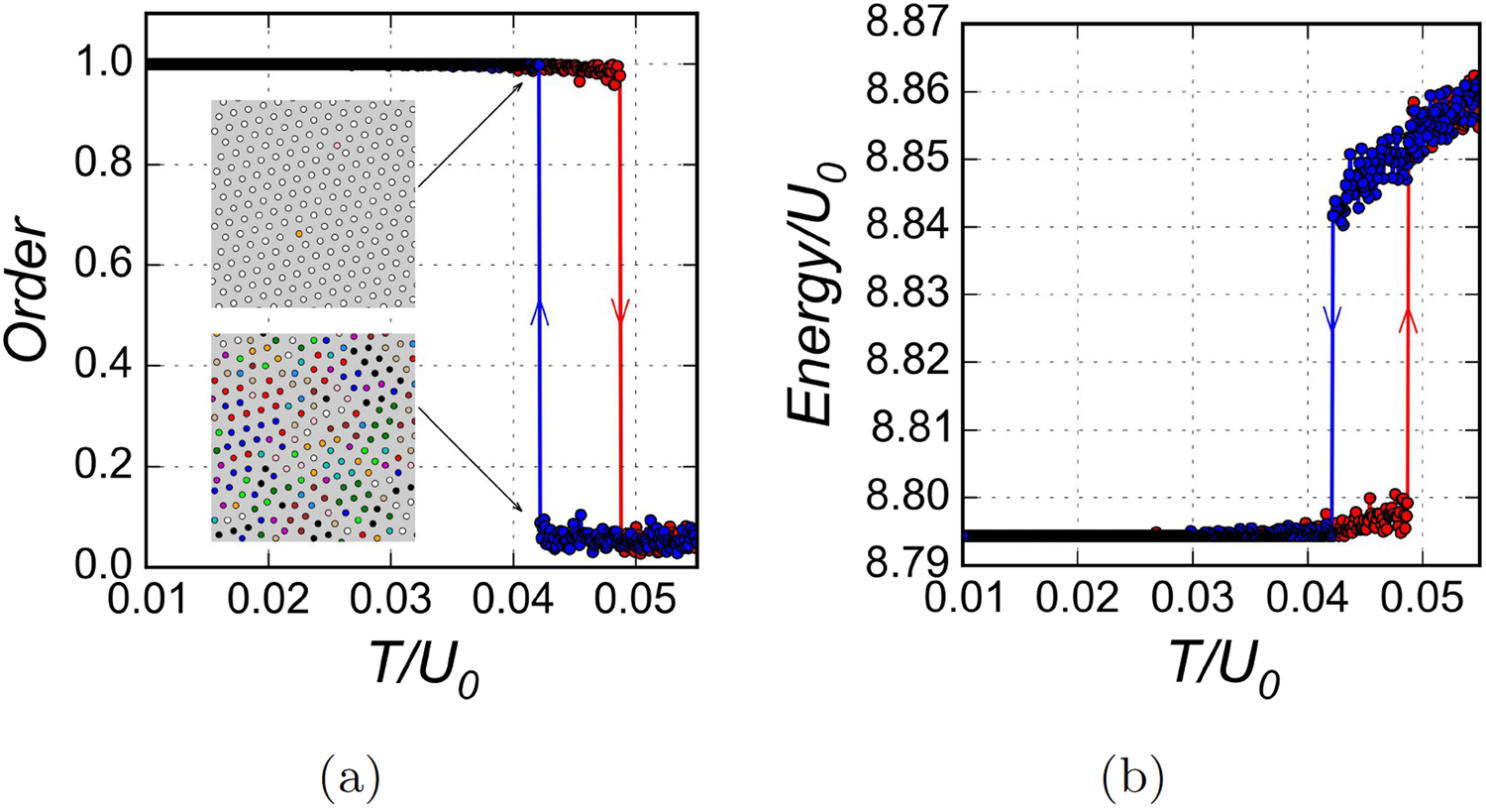
Temperature dependencies of integrated characteristics for the undoped system. (**a**) The order parameter; the insets show snapshots of configurations of the system just above and below the phase transition; (**b**) mean energy per particle. Blue symbols are for cooling and red symbols are for heating simulations.

On cooling, the order-disorder phase transition takes place at *T*_*c*_ ≈ 0.042*U*_0_, below which the triangular superlattice is formed confirming the expectations for the Wigner crystal. Temperature dependencies of the order parameter $M=\sqrt{13\sum {({m}_{i}-1/13)}^{2}/12}$, where *m*_*i*_ is the fraction of particles at the *i*-th sublattice (Fig. 3a) and of the mean value of energy per particle (Fig. 3b) indicate that the transition is of the first order. The insets in the Fig. 3a show a plenty of defects just above *T*_*c*_, while only two displaced positions are left just below *T*_*c*_.

On heating, the order-disorder phase transition takes place at *T* ≈ 0.049*U*_0_, which agrees with our mean field analysis (see Supplementary Sec. III). With increasing *l*_*s*_ the temperature hysteresis and the tendency to overcooling become more pronounced. An overcooling or even freezing into in a glass state feature is known for electronic systems with either a frozen disorder or a Coulomb frustrations^19^; however in the present model these both factors are absent – the effect is presumably due to only the long-range Coulomb interactions under the lattice constraints.

### Doped system

We emulate the doping (the charge injection) by seeding voids at random places and following the subsequent evolution. By global characterizations like those in Fig. 3, the order-disorder transition is preserved, while at a lower temperature. But locally the new mosaic ground state with the net of domain walls is formed as we will demonstrate below.

Seeding at *T* < *T*_*c*_ a small number of voids, down to two defects per sample, we observe that the single-void states are unstable with respect to their binding and progressive aggregation. Seeding more voids initiates their gradual coalescence into a globule of interconnected segments of domain walls. The resulting globule performs slowly a random diffusion over the sample while keeping closely its optimal shape and the structure of connections (the Supplementary Video of the system evolution under cooling and its description are presented in Supplementary Sec. I).

Despite it is not necessary to go through the order-disorder transition to obtain the globule structures–the seeding of voids can be performed within the ordered phase, but the equilibration times become much longer in this case. Therefore in order to obtain the low-temperature configurations for the doped systems we use the same strategy as for undoped ones: we cool down the system starting from the disordered state at *T* > *T*_*c*_.

**Figure 4:**
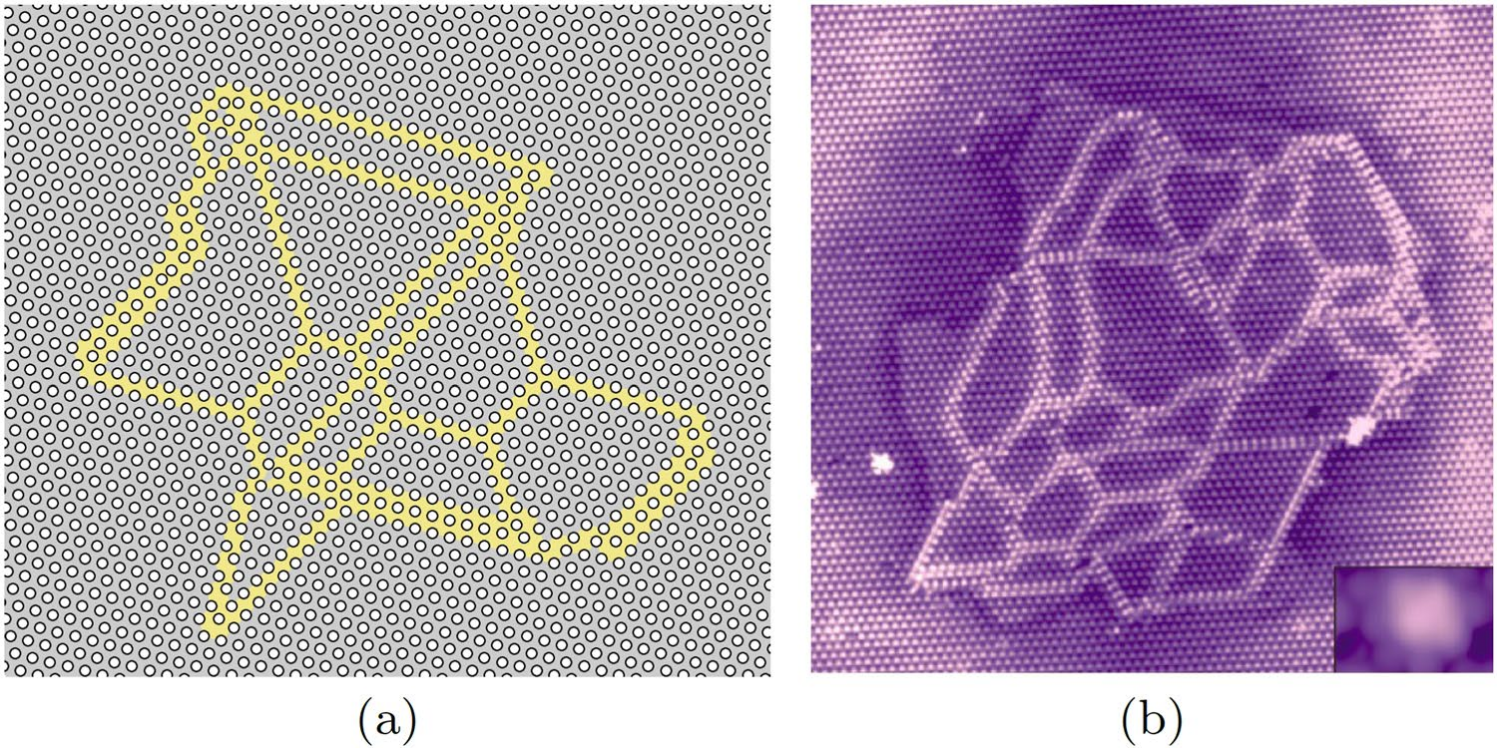
Globule structures. (**a**) The present modeling for *ν*_*voids*_ ≈ 0.9% in the domain walls representation, *T* = 0.01*U*_0_; (**b**) from experiments in^8^.

Figure 4a shows the low-*T* configuration of a 156 × 182 system, where initially *N*_*p*_ = 2184 of particles (with the the corresponding concentration of voids *ν*_*voids*_ ≈ 0.9%) were randomly seeded and then the system was slowly cooled from *T* = 0.055*U*_0_ > *T*_*c*_ down to *T* = 0.01*U*_0_. In spite of the initial random distribution of particles over the whole sample, finally the voids aggregate into a single globule immersed into a connected volume of the unperturbed crystal. We compare the results of our modeling in Fig. 4a with the experimental picture in Fig. 4b^8^. Similar patterns have been observed also in other experiments^6^: (Fig. 3 of the supplement) and^9^ (Fig. 1). By adding the particles to the obtained globule configuration, we can reverse the doping and anneal the system at *T* = 0.04*U*_0_ < *T*_*c*_. Then the globule gets erased and the system relaxes pretty fast to the uniform state, which takes $\sim 5\times {10}^{6}$ steps, see Supplementary Sec. II for the details.

**Figure 5:**
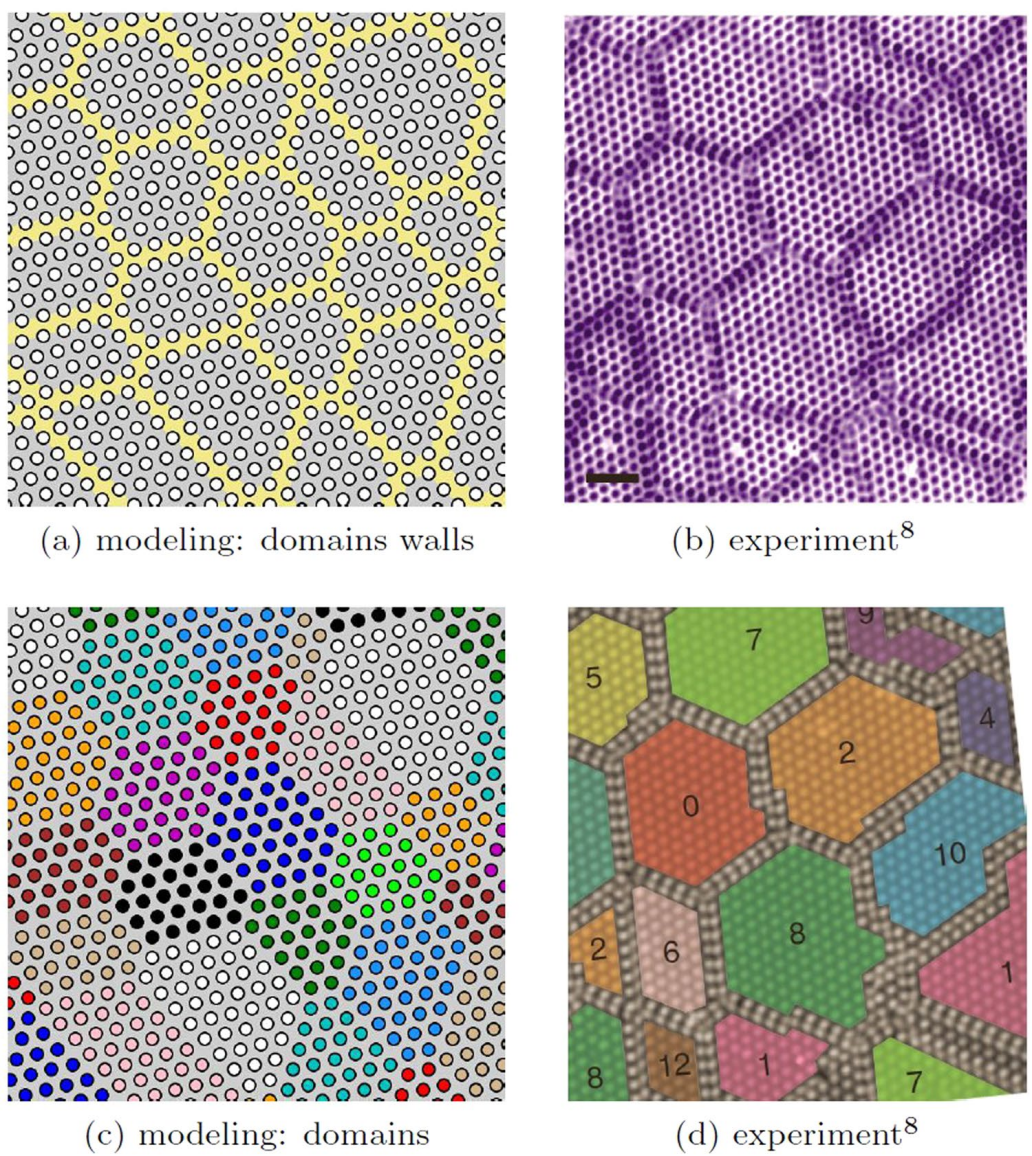
The modeling for a high doping (**a,c**) vs experiments (**b,d**). Maps of domain walls (**a,b**) and of domains (**c,d**). (**a,c**) Show the present modeling with *ν*_*voids*_ = 1.9% of voids at low temperature *T* = 0.01*U*_0_, the coloring scheme for domains is indicated in Fig. 1. (**b,d**) are adapted from^8^, numbers in (**d**) show the corresponding coloring scheme for domains.

With a further increase of doping, the globule size grows over the whole sample, and finally the branched net of domain walls divides the system into the mosaics of randomly shaped domains (Fig. 5a,c). The comparison of the modeling with the experiment on injection by the STM pulses is shown between panels (a) and (b) in Fig. 4, between panels (a) and (b), (c) and (d) in Fig. 5. The figures visualize a spectacular resemblance of our modeling with results from several STM experiments exploiting either the optical switching to the hidden state^5^ or the pulses from the STM tip^8,9^. Note that “irregular honeycomb network” structures were predicted for incommensurate phase of krypton on graphite with *ν*_0_ ≈ 1/3 and short-range interaction^20^, but with more topological restrictions than here.

## Discussion and Conclusions

Our simulations have shown an apparently surprising behavior: some effective attraction of voids develops from the purely repulsive Coulomb interactions. The coalescence of single voids starts already at their small concentration. For several voids seeded, we observe a gradual fusion of point defects into the globule of the domain walls. Increasingly branched net develops with augmenting of the voids concentration.

That can be understood indeed by noticing that the walls formation is not just gluing of voids but their fractionalization. The domain wall is fractionally (*q* = *ν*_0_*e*) charged per its crystal-unit length, thus reducing the Coulomb self-energy in comparison with the integer-charged single void. Being the charged objects, the domain walls repel each other, but as topological objects they can terminate only at branching points, thus forming in-plane globules. Their repulsion at adjacent layers meets no constraints, hence the experimentally observed alternation of the walls’ patterns among the neighboring layers^5,8,9^.

A similar while simpler doping induced phase transition to the state patterned by charged domain walls was predicted for quasi-1D electronic systems with 2-fold degeneracy from the Peierls effect of the spontaneous dimerization, see^21^.

Our modeling can be straightforwardly extended to other values of concentration *ν*_0_: like *ν*_0_ = 1/3 which is the minimal value where the pattern formation appears with qualitatively similar to the presented here results (see Supplementary Sec. IV) and corresponding, for example, to the $\sqrt{3}\times \sqrt{3}$ surface CDW observed in the lead coated germanium crystal^22^. For another experimentally known case of 2*H* − TaSe_2_ where *ν*_0_ = 1/9 the modeling results are quite different: in some doping range we see a “stripe” phase (see Supplementary Sec. IV), which indeed was experimentally observed in this material^23^. The exception of the case *ν*_0_ = 1/9 is rather natural, because here the basis vectors of the superlattice and of the underlying triangular lattice are parallel to each other, which allows for the existence of neutral elementary domain walls.

It is also possible to study the doping by electrons by seeding the interstitials rather than voids; here the new David stars substantially overlap with their neighbors giving rise to stronger lattice deformations, which may require for a more complicated model. The fragmentation with formation of walls is always confirmed while details of patterns can differ (see Supplementary Fig. 9).

The encouraging visual correspondence of our pictures with experimentally obtained patterns in different regimes of concentrations ensures a dominant role of the universal model.

## Methods

For numerical simulation of the classical lattice gas with interaction potential (1) we employed the Metropolis Monte Carlo method. We used the screening parameter *l*_*s*_ = 7.2*b* ≈ 2*a* and truncated the interactions to zero for sufficiently large interparticle distances–outside the hexagon with the side 40*b* (we also performed the modeling for *l*_*s*_ = 4.5*b* ≈ 1.25*a* with qualitatively similar results, see Supplementary Materials). At each temperature we performed ~5–10 millions of Monte Carlo steps depending on the numerical experiment. Temperature was linearly lowered from *T* = 0.055*U*_0_ down to *T* = 0.01*U*_0_ with a step Δ*T* = −0.0001*U*_0_. The following system sizes and numbers of particles were chosen: size 91 × 104 and *N*_*p*_ = 728 for undoped system; size 156 × 182 and *N*_*p*_ = 2184, *ν*_*voids*_ ≈ 0.9% for the globule system (Fig. 4a); size 142 × 168 and *N*_*p*_ = 1758, *ν*_*voids*_ ≈ 1.9% for the net system (Fig. 5a,c). Periodic rectangular boundary conditions were imposed.

### Data availability

The datasets generated during and analysed during the current study are available from the corresponding author on reasonable request.

## Electronic supplementary material

Supplementary material

Video - merging of 4 holes and diffusion
