# Supplementary material for: Modeling of networks and globules of charged domain walls observed in pump and pulse induced states
Source: Sci Rep. 2018 Mar 6;8:4043. doi: 10.1038/s41598-018-22308-7 (PMC5840135; doi:10.1038/s41598-018-22308-7)
Supplement: Supplementary file 1 — Supplementary material [file 41598_2018_22308_MOESM1_ESM.pdf]

Supplemental Material.
Modeling of networks and globules of charged domain walls observed in pump and pulse induced states.

Petr Karpov[1,*] and Serguei Brazovskii[1,2,3]

[1]*National University of Science and Technology "MISiS", Moscow, Russia*

[2]*CNRS UMR 8626 LPTMS, University of Paris-Sud, University of Paris-Saclay, Orsay, France*

[3]*Jozef Stefan Institute, Jamova 39, SI-1000 Ljubljana, Slovenia*

(Dated: January 27, 2018)

I. MOVIE: COALESCENCE OF VOIDS AND CLUSTER DIFFUSION

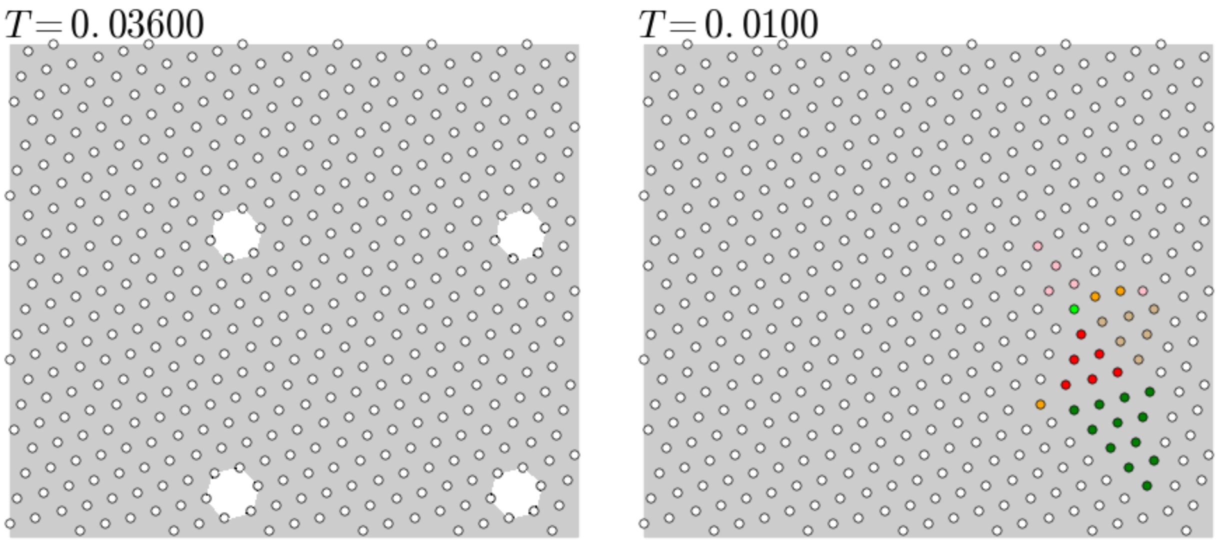

(a) (b)

FIG. 1. (a) The initial position of the simulation at $T = 0.036U_0$, holes' locations are highlighted by white background; (b) the final position of the simulation at $T = 0.010U_0$.

The supplementary movie illustrates the process of Monte Carlo (MC) cooling of the system, after several voids have been added to the ordered state below the transition temperature (here $l_s = 1.25a$). This procedure differs from the one exploited to obtain the final states reported in the main text. There we always started from a random disordered state at $T > T_c$ in order to reach a faster equilibration. The movie demonstrates two regimes in the course of the cooling: (1) merging of voids into a single big cluster seen as the globule of domain walls; (2) a subsequent random diffusion of the cluster.

We start the simulation from the ordered system where 4 voids have been seeded by hands. (Fig. 1a shows the initial position) and cool it from $T = 0.036U_0$ down to $T = 0.010U_0$ with step $\Delta T = -0.0001U_0$, making at each temperature 10000 MC steps for higher temperatures ($T \geq 0.0310U_0$) or 50000 MC steps for lower temperatures ($T < 0.0310U_0$).

First, each of 4 voids very quickly disintegrates into a small cluster – a "mini-globule" ("1-cluster"), so, within the studied model individual voids are unstable; in the real experiments void are presumably stabilized additional short-range elastic interactions.

Then a random diffusion begins (note, that periodical boundary conditions are imposed).

Being the charged objects, the 1-clusters are repelled from each other. Nevertheless, if two 1-clusters overcome a potential barrier, they merge into a stable 2-cluster, this which lowers the system energy (the qualitative explanation of this counterintuitive effect is given in the main body of the paper). The first event of two 1-clusters merging happens at $T = 0.03548U_0$ after ~ 52000 MC steps (on the 5-th second of the video).

After several collisions and unsuccessful coalescence attempts, the 2-cluster merges with a 1-cluster at $T = 0.03407U_0$ (after ~ 193000 MC steps, 19-th second of the video). Final merging to a single cluster happens shortly after that at $T = 0.03378U_0$.

Afterwards, the resultant big cluster performs a random diffusion through the sample. After the 50-th second, the video is speeded up by the factor of 50. Figure 1b shows the final position of the simulation.

We conclude that it is indeed energetically favourable and dynamically sustainable for separate holes to merge into a single cluster, in other words the walls globule.

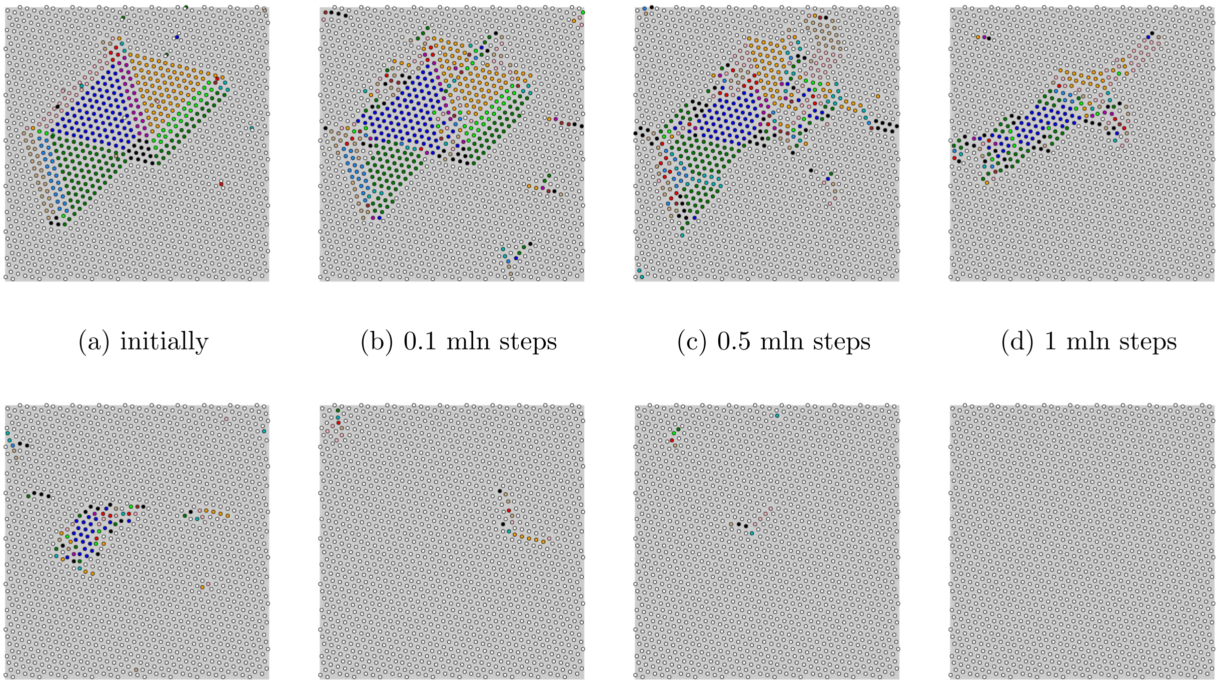

(a) initially (b) 0.1 mln steps (c) 0.5 mln steps (d) 1 mln steps

(e) 2 mln steps (f) 3 mln steps (g) 4 mln steps (h) 5 mln steps

FIG. 2. (a) Configuration of the initially prepared globule system, where interstitial atoms were introduced "by hands"; (b-h) configurations after the subsequent MC evolution at $T = 0.04U_0 < T_c$. Screening length $l_s = 2a$.

II. CURING A GLOBULE

In this section we simulate the erasing of a globule after the doping is reversed. We take an initial low-temperature globule configuration (which is close to the ground state for the given value of doping) and then seed "by hands" interstitial particles at random places, making the system globally undoped. The results presented in Fig. 2 show the gradual curing of the globule and relaxation to the ground state.

We believe that a process analogous to the shown one is unlikely to happen in the real system, because the local energy of the interstitial configuration is very high (compared to the domain wall energy). Moreover, in order to cure such a globule, positions of almost all polarons inside the globule have to be readjusted (not only the in-wall polarons), which makes the energy barrier – between the globule metastable state and the uniform ground state – proportional to the area of the globule.

III. ORDER-DISORDER PHASE TRANSITION: MEAN FIELD THEORY

In this section we present the mean field theory for the order-disorder phase transition for an undoped system. Since the interactions are of the long-range nature and many particles affect the given one, we expect mean-filed theory to be a good approximation, at least for determining the transition temperature.

At $T = 0$, the perfectly ordered state is observed, where all particles occupy the same sublattice (one of the 13 equivalent sublattices, not counting for the mirror symmetry, as discussed in the main text). With gradually increasing temperature, at some critical one $T = T_c$, the order momentarily breaks.

In order to determine T_c we consider a simplified version of the model, dividing the whole system into closely packed David stars, and allowing for each particle to occupy only one of 13 sites of its David star (Fig. 3; the ground state at $T = 0$ is reached if only the central sites are occupied.). This simplification will lead to a slight overestimation for T_c.

Since the activation energy Δ_1 to excite a particle from its regular position in the David star center to the first coordination sphere is several times less than the analogous energy for the second coordination sphere Δ_2 (for example, for the studied in the main text case $l_s = 7.2b$ we can numerically find $\Delta_1 \approx 0.346U_0$, $\Delta_2 \approx 1.547U_0$). Then taking into account

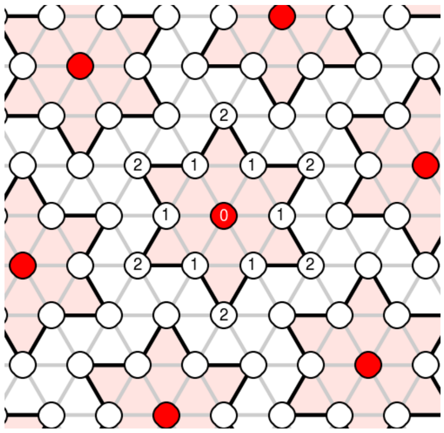

FIG. 3. One of the ground states of the system, where particles (red circles) occupy the same sublattice. 1-st, and 2-nd coordination spheres for a given particle ("0") are shown.

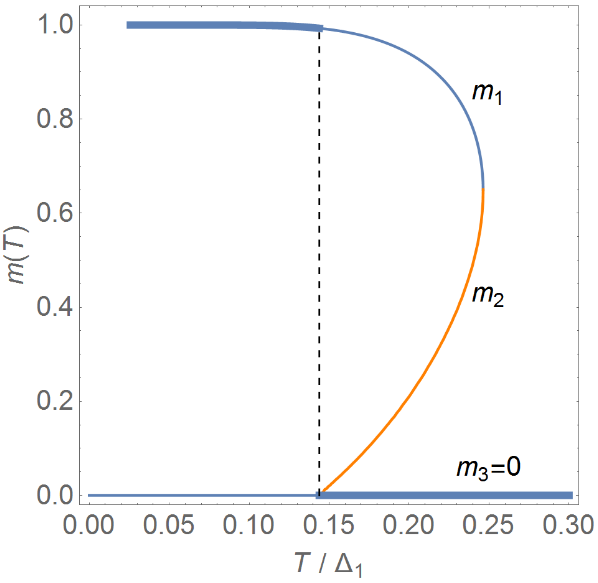

FIG. 4. Three branches $m(T)$ of the solution of the self-consistency equation (2). Solutions m_1 and m_3 (blue) correspond to the local minima of the free energy; their stable branches are shown in bold. Solution m_2 (orange) corresponds to the local maximum of the free energy. Dashed line shows the critical temperature $T_c = \Delta_1/7$.

only the 1-st coordination sphere seems to be a reasonable first approximation and we arrive at a simpler model with only 7 possible states for each particle.

Consider a particle in the mean field of other particles. Let (s_0, s_1) be the occupation numbers of the 0-th and the 1-st coordination spheres respectively. In the ground state we

have $s_0 = 1$, $s_1 = 0$; in the excited state $s_0 = 0$, $s_1 = 1$. Introduce the order parameter

$$m = \frac{7}{6}\langle s_0 - \frac{1}{7}\rangle, \tag{1}$$

so that in the ordered phase $m = 1$, in the disordered phase $m = 0$. The activation energy for a given particle to be excited from the central cite is proportional to the number of particles at the central-cite sublattice; so we write this temperature-dependent activation energy as $\Delta(T) = \Delta_1 \cdot m(T)$. The partition function is $Z(T) = 1 + 6e^{-\Delta_1 m/T}$. From the self-consistency condition (1), we get $m = \left(1 - \frac{7}{6}e^{-\Delta_1 m/T}\right)/Z$ or

$$m = \frac{1 - e^{-m\Delta_1/T}}{1 + 6e^{-m\Delta_1/T}} \tag{2}$$

Solutions of this self-consistency equation are shown in Fig. 4. Expanding RHS of (2) to the first order in m we get $m = m\Delta_1/7T + o(m)$, which yields to the critical temperature

$$T_c = \frac{\Delta_1}{7}. \tag{3}$$

For $l_s = 7.2b$ we have $\Delta_1 \approx 0.346U_0$, and formula (3) gives us $T_c \approx 0.0494U_0$, which is very successfully compared with the MC simulation result on heating: $T_c \approx 0.049U_0$. For $l_s = 4.5b$ we have $\Delta_1 \approx 0.442U_0$, so the mean-field result is $T_c \approx 0.0631U_0$; the MC simulation on heating gives: $T_c \approx 0.063U_0$.

We conclude that the mean field theory is in a very good agreement (within $\sim 1\%$) with the results of the MC simulation. This is rather natural because for the screened Coulomb long-range interaction many coordination spheres of particle's neighbors contribute to the mean field for a given particle (this fact is incorporated into the definition of the excitation energy Δ_1). The triple branching of the order parameter (Fig. 4) signifies the first order phase transition in accordance with both simulations (cf. Fig. 3a of the main text) and experiments – this ordering phase transition may be considered as a "cartoon" version of the CDW transition with formation of an electronic crystal from a uniform state.

IV. RESULTS FOR DIFFERENT VALUES OF CONCENTRATION ν_0

In this section we, first, give the classification of filling factors on a triangular lattice, and then analyze some representative cases. Figure 5 shows ordinal numbers of neighbors for a given particle (black) – 1 corresponds to the nearest neighbor, 2 to the next-nearest

neighbor etc. Knowledge of where the particle's nearest neighbor sits determines the whole superstructure. Table I presents the correspondence between the number of the occupied sublattice and the concentration of the particles. The third column of the table shows, whether the superlattice possesses only charged domain walls or not: this is connected to the fact, whether the basis vectors of the superlattice are parallel to the basis vectors of the underlying triangular lattice. This is the key factor, determining the domain walls' patterns in doped systems. The forth column shows, whether the superlattice possesses two different sectors of ground states, which can not be obtained one from another by only a translation – in this case the mirror symmetry is also needs to be broken.

In the further subsections we briefly discuss several experimentally accessible cases, with the concentrations $\nu_0 = 1/3$, $1/9$, or $1/13$.

TABLE I. Classification of commensurate filling factors on a triangular lattice

# of neighbor	concentration ν_0	only charged domain walls?	two mirror symmetric sectors?
2	1/3	yes	no
3	1/4	no	no
4	1/7	yes	yes
5	1/9	no	no
6	1/12	yes	no
7	1/13	yes	yes
8	1/16	no	no
9	1/19	yes	yes
10	1/21	yes	yes

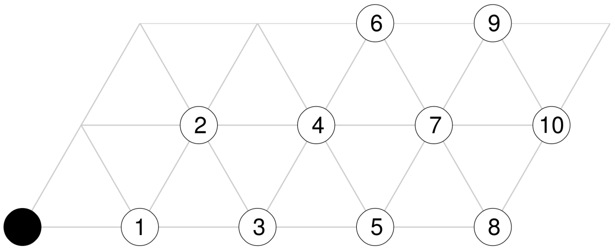

FIG. 5. Neighbors of a given site (black) with successively increasing distances from it.

A. $\nu_0 = 1/3$

Concentration $\nu_0 = 1/3$ is the minimal case, where we observe qualitatively similar patterns as for $\nu_0 = 1/13$. There is, however, an important distinction: single holes are much more stable here and their threshold concentration is necessary to initiate a globule creation. This case corresponds, for example, to charge density waves in monolayers of Pb on the surface of Ge.[1].

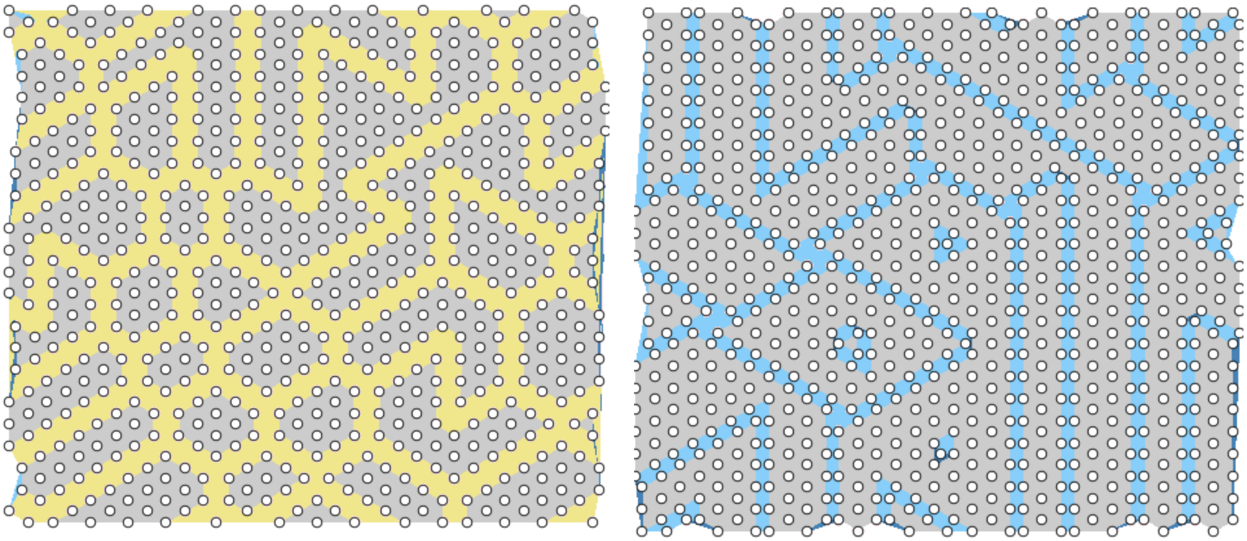

(a) (b)

FIG. 6. $\nu_0 = 1/3$: domain walls representation for (a) positive doping; (b) negative doping.

B. $\nu_0 = 1/9$

In the case $\nu_0 = 1/9$ the minimal domain walls are neutral, because unit vectors of the superlattice and of the underlying triangular lattice are parallel to each other for this concentration, so the results are qualitatively different from 1/3 and 1/13 cases (Fig. 7). Which connectivity is more favorable (stripes or a network) depends on the sign of wall-crossing energy[2]. Interestingly the "stripe" phase is experimentally observed on heating of $\nu_0 = 1/9$ commensurate charge density wave in $2H - \mathrm{TaSe}_2$, see[3].

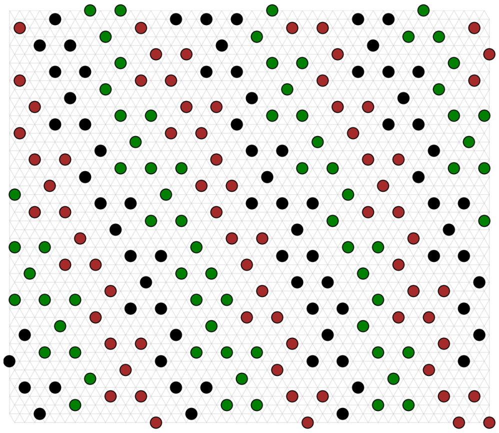

FIG. 7. $\nu_0 = 1/9$: domains representation for the “stripe” phase for with positive doping. Here we use 9-coloring scheme (only 3 of them appear on the figure), analogous to 13-coloring scheme used in the main text.

C. $\nu_0 = 1/13$

The case of positive doping and $l_s = 2a$ was considered in the main text; here we present the results for another value of the screening length $l_s = 1.25a$ (Fig. 8), with qualitatively similar results.

Here we also extend the results of the main text to the case of the electrons' doping which accumulate into interstitials rather than voids (Fig. 9). For negative doping we still observe the charge fractionalization phenomena and qualitatively similar to the case of positive doping structures. The wall crossings are still favorable (thus no “stripe phase”), but the connection of 4 walls becomes also favorable as the one of 3 walls, which is presumably governed by the short-range physics or the choice of the boundary conditions. Because of this, visually the pictures look differently. We expect that for the higher values of l_s and larger systems (when boundary conditions become less important), the metastable states with the lowest lying energies will represent the “irregular honeycomb networks”[4] as observed for the positive doping.

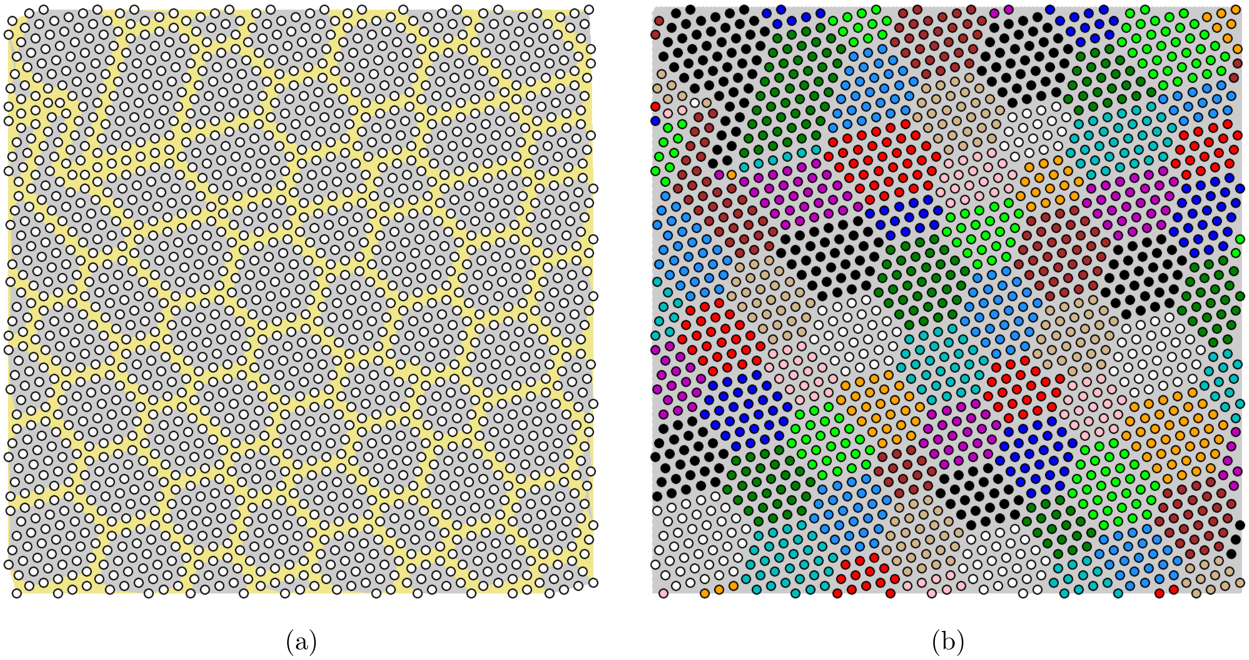

FIG. 8. $\nu_0 = 1/13$: net structure for positive doping, $\nu_{voids} = 1.9\%$, $l_s = 1.25a$, $T = 0.01U_0$. (a) Domain wall representation; (b) domain representation.

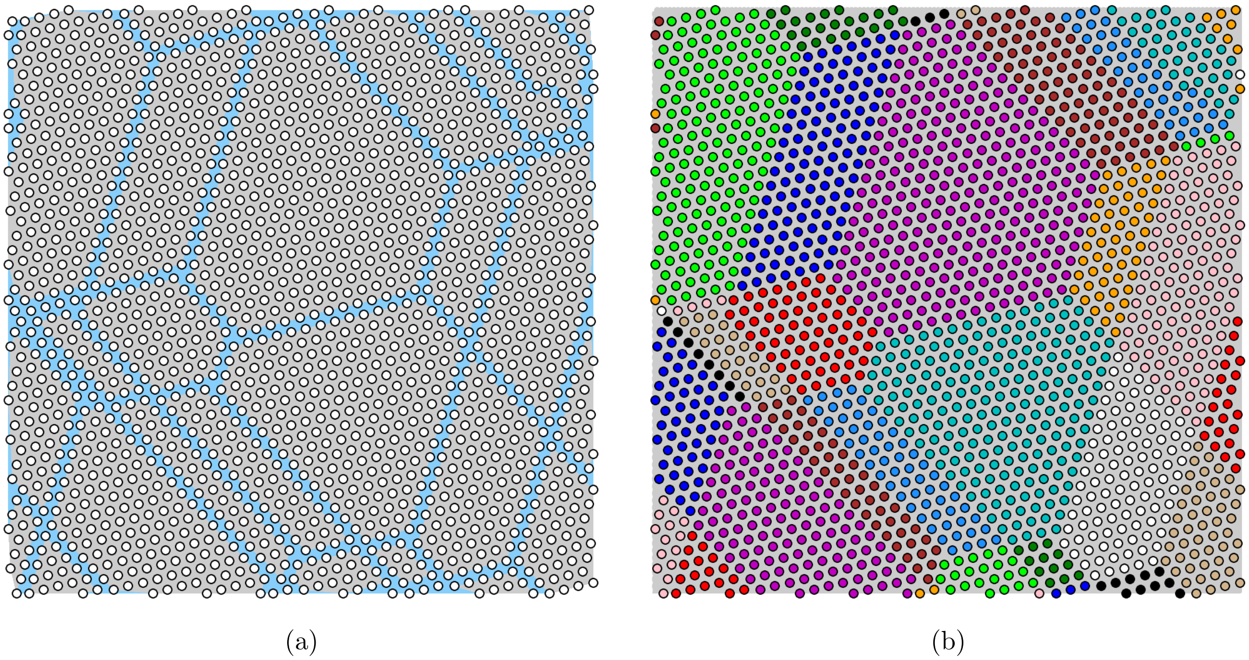

FIG. 9. $\nu_0 = 1/13$: net structure for negative doping, $\nu_{interst} \approx 1.5\%$, $l_s = 1.25a$, $T = 0.01U_0$: (a) domain wall representation; (b) domain representation.

V. TWO CHIRALITIES

As it is mentioned in the main text, the ground state possesses not only 13-fold degeneracy associated with translations of the superlattice with respect to the underlying lattice, but also an additional 2-fold degeneracy associated with the chiral (mirror) symmetry. This leads to existence of two mirror-symmetric phases with different chiralities (Fig. 10).

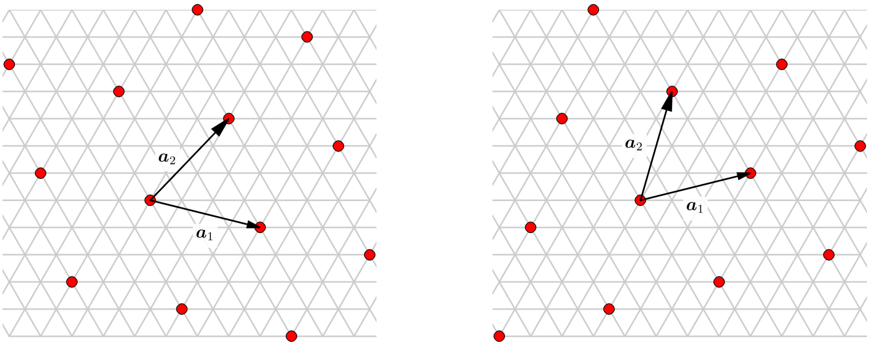

FIG. 10. Ground states of with two different chiralities: (a) left; (b) right.

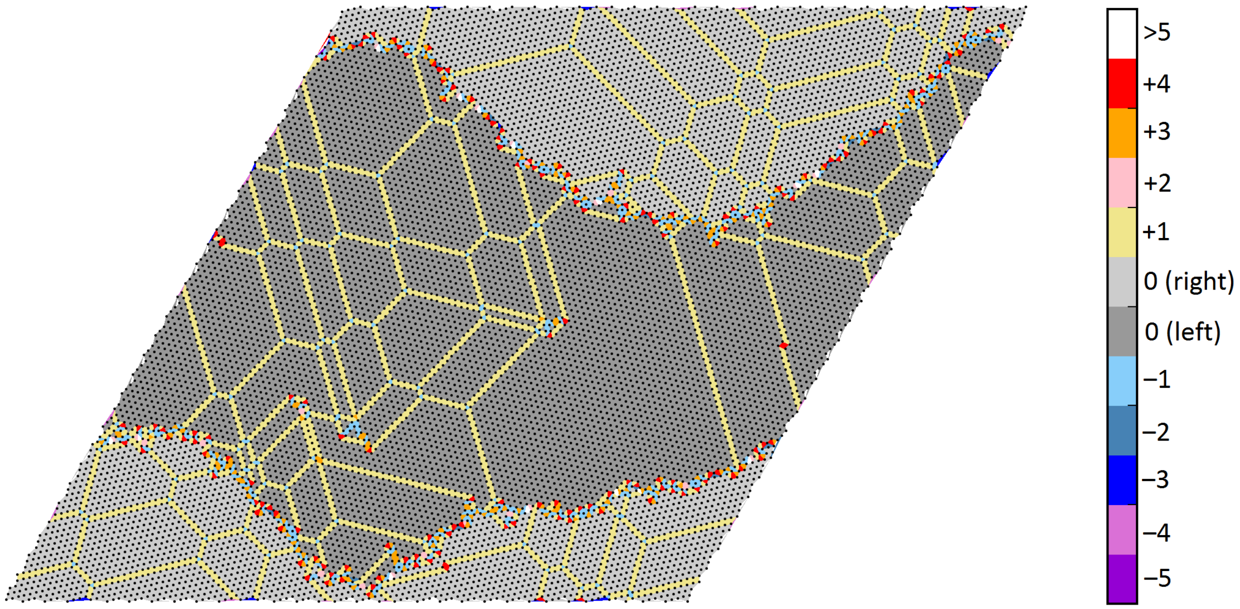

FIG. 11. Domains with different chiralities are shown with lighter and darker shades of gray color. The color scheme is for types of domain walls (or more precisely for the areas of triangles in the Delaunay triangulation). $\nu_{voids} = 1.2\%$, $l_s = 1.25a$, $T = 0.02U_0$.

If annealing is performed sufficiently fast – much faster than for the simulations described

in the previous supplementary sections and in the main text (or if the model system is sufficiently large), then coexistent domains with two different chiralities can be observed. The corresponding domain wall – twin grain boundary – is relatively high-energetic, therefore under the annealing procedure such walls disappear rather fast. Fig. 11 shows configuration of the system 399×399 doped by 1.2% of voids annealed down to $T = 0.02U_0$, where domains with two different chiralities are observed.

---

* karpov.petr@gmail.com

[1] J. M. Carpinelli, H. H. Weitering, E. W. Plummer, and R. Stumpf, Direct observation of a surface charge density wave. *Nature* **381**, 398-400 (1996).

[2] P. Bak, Commensurate phases, incommensurate phases and the devil's staircase. *Rep. Prog. Phys.* **45**, 587-629 (1982).

[3] R. M. Fleming, D. E. Moncton, D. B. McWhan, and F. J. DiSalvo, Broken Hexagonal Symmetry in the Incommensurate Charge-Density Wave Structure of $2H - \mathrm{TaSe_2}$. *Phys. Rev. Lett.* **45**, 576 (1980).

[4] J. Villain, Commensurate-incommensurate transition of krypton monolayers on graphite: a low temperature theory. *Surface Science* **97**, 219-242 (1980).
